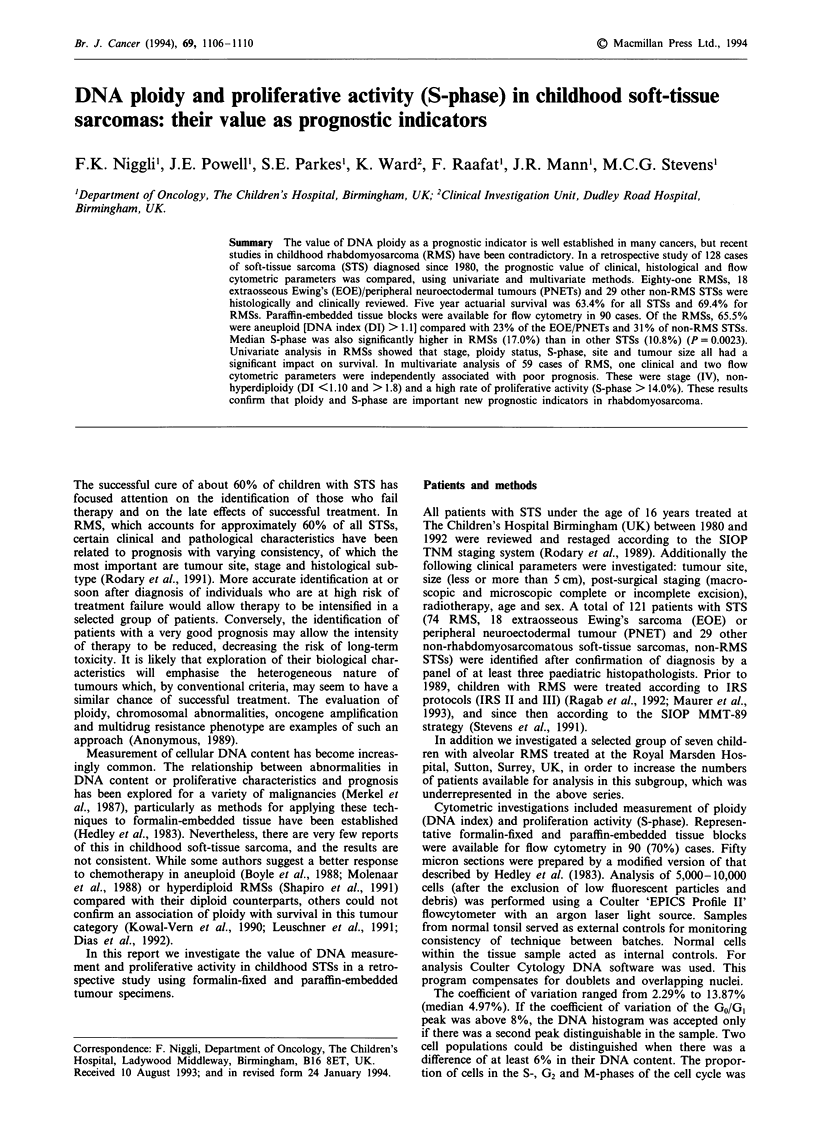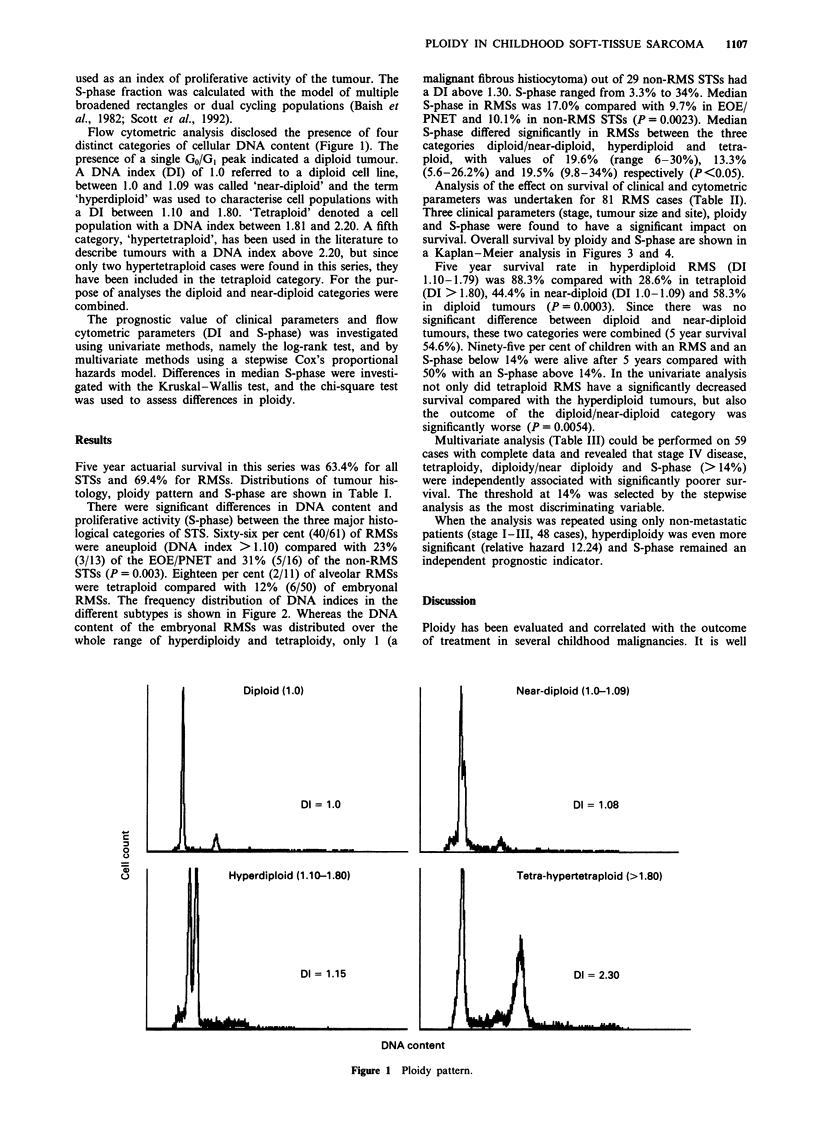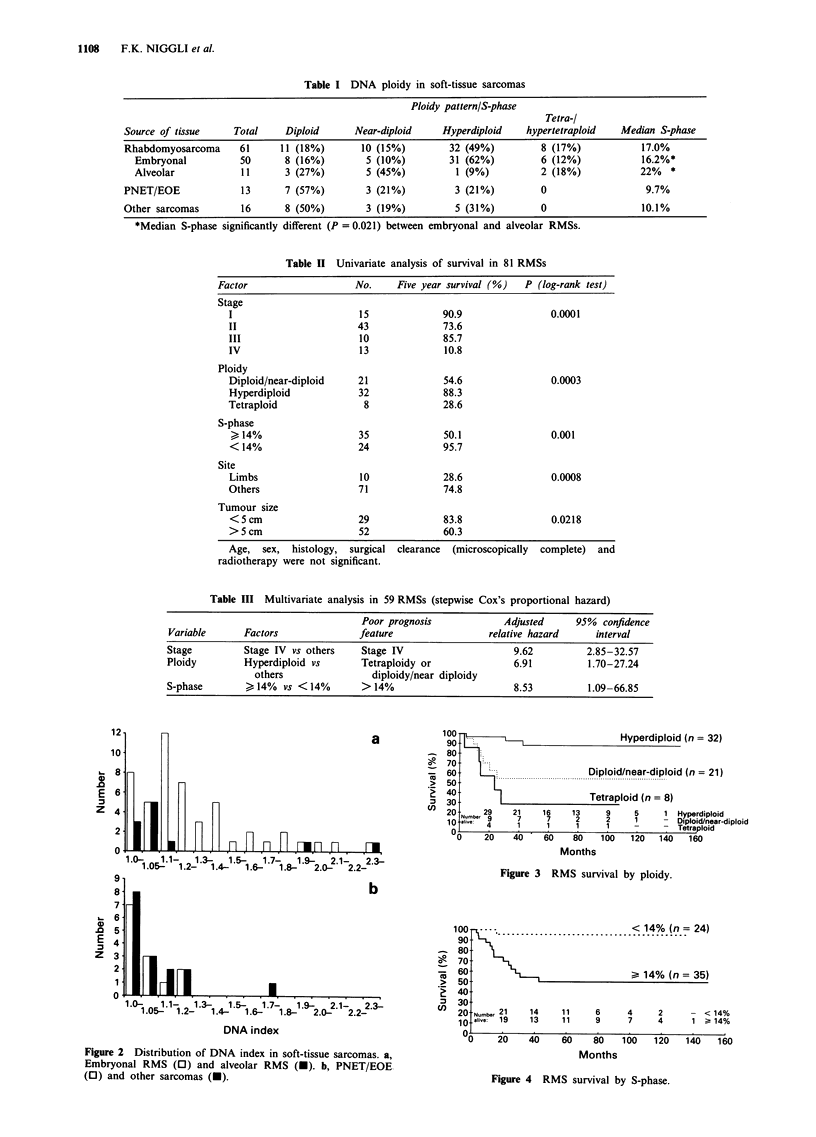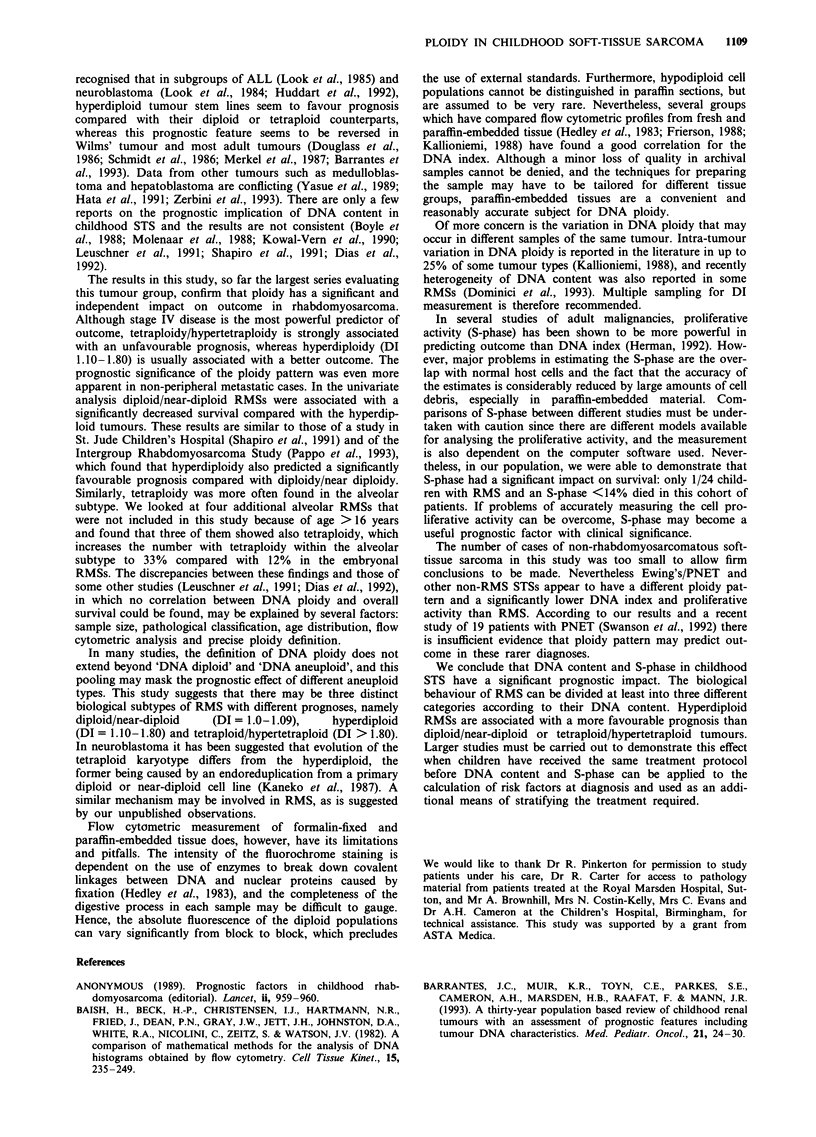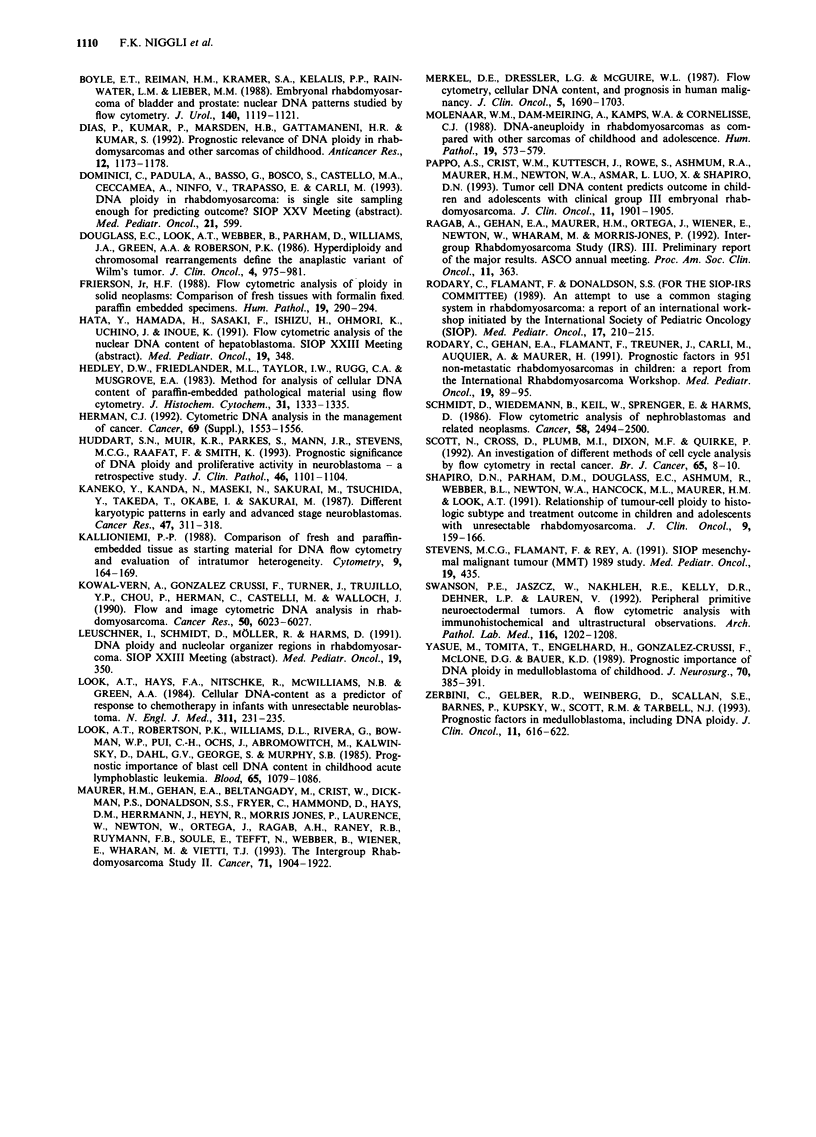# DNA ploidy and proliferative activity (S-phase) in childhood soft-tissue sarcomas: their value as prognostic indicators.

**DOI:** 10.1038/bjc.1994.217

**Published:** 1994-06

**Authors:** F. K. Niggli, J. E. Powell, S. E. Parkes, K. Ward, F. Raafat, J. R. Mann, M. C. Stevens

**Affiliations:** Department of Oncology, Children's Hospital, Birmingham, UK.

## Abstract

The value of DNA ploidy as a prognostic indicator is well established in many cancers, but recent studies in childhood rhabdomyosarcoma (RMS) have been contradictory. In a retrospective study of 128 cases of soft-tissue sarcoma (STS) diagnosed since 1980, the prognostic value of clinical, histological and flow cytometric parameters was compared, using univariate and multivariate methods. Eighty-one RMSs, 18 extraosseous Ewing's (EOE)/peripheral neuroectodermal tumours (PNETs) and 29 other non-RMS STSs were histologically and clinically reviewed. Five year actuarial survival was 63.4% for all STSs and 69.4% for RMSs. Paraffin-embedded tissue blocks were available for flow cytometry in 90 cases. Of the RMSs, 65.5% were aneuploid [DNA index (DI) > 1.1] compared with 23% of the EOE/PNETs and 31% of non-RMS STSs. Median S-phase was also significantly higher in RMSs (17.0%) than in other STSs (10.8%) (P = 0.0023). Univariate analysis in RMSs showed that stage, ploidy status, S-phase, site and tumour size all had a significant impact on survival. In multivariate analysis of 59 cases of RMS, one clinical and two flow cytometric parameters were independently associated with poor prognosis. These were stage (IV), nonhyperdiploidy (DI < 1.10 and > 1.8) and a high rate of proliferative activity (S-phase > 14.0%). These results confirm that ploidy and S-phase are important new prognostic indicators in rhabdomyosarcoma.


Br. J. Cancer (1994), 69, 1106-1110 © Macmillan Press Ltd., 1994

### DNA ploidy and proliferative activity (S-phase) in childhood soft-tissue sarcomas: their value as prognostic indicators

F.K. Niggli[1], J.E. Powell[1], S.E. Parkes[1], K. Ward[2], F. Raafat[1], J.R. Mann[1], M.C.G. Stevens[1]

[1]*Department of Oncology, The Children's Hospital, Birmingham, UK;* [2]*Clinical Investigation Unit, Dudley Road Hospital, Birmingham, UK.*

**Summary** The value of DNA ploidy as a prognostic indicator is well established in many cancers, but recent studies in childhood rhabdomyosarcoma (RMS) have been contradictory. In a retrospective study of 128 cases of soft-tissue sarcoma (STS) diagnosed since 1980, the prognostic value of clinical, histological and flow cytometric parameters was compared, using univariate and multivariate methods. Eighty-one RMSs, 18 extraosseous Ewing's (EOE)/peripheral neuroectodermal tumours (PNETs) and 29 other non-RMS STSs were histologically and clinically reviewed. Five year actuarial survival was 63.4% for all STSs and 69.4% for RMSs. Paraffin-embedded tissue blocks were available for flow cytometry in 90 cases. Of the RMSs, 65.5% were aneuploid [DNA index (DI) > 1.1] compared with 23% of the EOE/PNETs and 31% of non-RMS STSs. Median S-phase was also significantly higher in RMSs (17.0%) than in other STSs (10.8%) ($P = 0.0023$). Univariate analysis in RMSs showed that stage, ploidy status, S-phase, site and tumour size all had a significant impact on survival. In multivariate analysis of 59 cases of RMS, one clinical and two flow cytometric parameters were independently associated with poor prognosis. These were stage (IV), non-hyperdiploidy (DI <1.10 and > 1.8) and a high rate of proliferative activity (S-phase > 14.0%). These results confirm that ploidy and S-phase are important new prognostic indicators in rhabdomyosarcoma.

The successful cure of about 60% of children with STS has focused attention on the identification of those who fail therapy and on the late effects of successful treatment. In RMS, which accounts for approximately 60% of all STSs, certain clinical and pathological characteristics have been related to prognosis with varying consistency, of which the most important are tumour site, stage and histological subtype (Rodary *et al.*, 1991). More accurate identification at or soon after diagnosis of individuals who are at high risk of treatment failure would allow therapy to be intensified in a selected group of patients. Conversely, the identification of patients with a very good prognosis may allow the intensity of therapy to be reduced, decreasing the risk of long-term toxicity. It is likely that exploration of their biological characteristics will emphasise the heterogeneous nature of tumours which, by conventional criteria, may seem to have a similar chance of successful treatment. The evaluation of ploidy, chromosomal abnormalities, oncogene amplification and multidrug resistance phenotype are examples of such an approach (Anonymous, 1989).

Measurement of cellular DNA content has become increasingly common. The relationship between abnormalities in DNA content or proliferative characteristics and prognosis has been explored for a variety of malignancies (Merkel *et al.*, 1987), particularly as methods for applying these techniques to formalin-embedded tissue have been established (Hedley *et al.*, 1983). Nevertheless, there are very few reports of this in childhood soft-tissue sarcoma, and the results are not consistent. While some authors suggest a better response to chemotherapy in aneuploid (Boyle *et al.*, 1988; Molenaar *et al.*, 1988) or hyperdiploid RMSs (Shapiro *et al.*, 1991) compared with their diploid counterparts, others could not confirm an association of ploidy with survival in this tumour category (Kowal-Vern *et al.*, 1990; Leuschner *et al.*, 1991; Dias *et al.*, 1992).

In this report we investigate the value of DNA measurement and proliferative activity in childhood STSs in a retrospective study using formalin-fixed and paraffin-embedded tumour specimens.

Correspondence: F. Niggli, Department of Oncology, The Children's Hospital, Ladywood Middleway, Birmingham, B16 8ET, UK.
Received 10 August 1993; and in revised form 24 January 1994.

### Patients and methods

All patients with STS under the age of 16 years treated at The Children's Hospital Birmingham (UK) between 1980 and 1992 were reviewed and restaged according to the SIOP TNM staging system (Rodary *et al.*, 1989). Additionally the following clinical parameters were investigated: tumour site, size (less or more than 5 cm), post-surgical staging (macroscopic and microscopic complete or incomplete excision), radiotherapy, age and sex. A total of 121 patients with STS (74 RMS, 18 extraosseous Ewing's sarcoma (EOE) or peripheral neuroectodermal tumour (PNET) and 29 other non-rhabdomyosarcomatous soft-tissue sarcomas, non-RMS STSs) were identified after confirmation of diagnosis by a panel of at least three paediatric histopathologists. Prior to 1989, children with RMS were treated according to IRS protocols (IRS II and III) (Ragab *et al.*, 1992; Maurer *et al.*, 1993), and since then according to the SIOP MMT-89 strategy (Stevens *et al.*, 1991).

In addition we investigated a selected group of seven children with alveolar RMS treated at the Royal Marsden Hospital, Sutton, Surrey, UK, in order to increase the numbers of patients available for analysis in this subgroup, which was underrepresented in the above series.

Cytometric investigations included measurement of ploidy (DNA index) and proliferation activity (S-phase). Representative formalin-fixed and paraffin-embedded tissue blocks were available for flow cytometry in 90 (70%) cases. Fifty micron sections were prepared by a modified version of that described by Hedley *et al.* (1983). Analysis of 5,000-10,000 cells (after the exclusion of low fluorescent particles and debris) was performed using a Coulter 'EPICS Profile II' flowcytometer with an argon laser light source. Samples from normal tonsil served as external controls for monitoring consistency of technique between batches. Normal cells within the tissue sample acted as internal controls. For analysis Coulter Cytology DNA software was used. This program compensates for doublets and overlapping nuclei.

The coefficient of variation ranged from 2.29% to 13.87% (median 4.97%). If the coefficient of variation of the $G_0/G_1$ peak was above 8%, the DNA histogram was accepted only if there was a second peak distinguishable in the sample. Two cell populations could be distinguished when there was a difference of at least 6% in their DNA content. The proportion of cells in the S-, $G_2$ and M-phases of the cell cycle was used as an index of proliferative activity of the tumour. The S-phase fraction was calculated with the model of multiple broadened rectangles or dual cycling populations (Baish *et al.*, 1982; Scott *et al.*, 1992).

Flow cytometric analysis disclosed the presence of four distinct categories of cellular DNA content (Figure 1). The presence of a single $G_0/G_1$ peak indicated a diploid tumour. A DNA index (DI) of 1.0 referred to a diploid cell line, between 1.0 and 1.09 was called 'near-diploid' and the term 'hyperdiploid' was used to characterise cell populations with a DI between 1.10 and 1.80. 'Tetraploid' denoted a cell population with a DNA index between 1.81 and 2.20. A fifth category, 'hypertetraploid', has been used in the literature to describe tumours with a DNA index above 2.20, but since only two hypertetraploid cases were found in this series, they have been included in the tetraploid category. For the purpose of analyses the diploid and near-diploid categories were combined.

The prognostic value of clinical parameters and flow cytometric parameters (DI and S-phase) was investigated using univariate methods, namely the log-rank test, and by multivariate methods using a stepwise Cox's proportional hazards model. Differences in median S-phase were investigated with the Kruskal–Wallis test, and the chi-square test was used to assess differences in ploidy.

### Results

Five year actuarial survival in this series was 63.4% for all STSs and 69.4% for RMSs. Distributions of tumour histology, ploidy pattern and S-phase are shown in Table I.

There were significant differences in DNA content and proliferative activity (S-phase) between the three major histological categories of STS. Sixty-six per cent (40/61) of RMSs were aneuploid (DNA index $>1.10$) compared with 23% (3/13) of the EOE/PNET and 31% (5/16) of the non-RMS STSs ($P = 0.003$). Eighteen per cent (2/11) of alveolar RMSs were tetraploid compared with 12% (6/50) of embryonal RMSs. The frequency distribution of DNA indices in the different subtypes is shown in Figure 2. Whereas the DNA content of the embryonal RMSs was distributed over the whole range of hyperdiploidy and tetraploidy, only 1 (a malignant fibrous histiocytoma) out of 29 non-RMS STSs had a DI above 1.30. S-phase ranged from 3.3% to 34%. Median S-phase in RMSs was 17.0% compared with 9.7% in EOE/PNET and 10.1% in non-RMS STSs ($P = 0.0023$). Median S-phase differed significantly in RMSs between the three categories diploid/near-diploid, hyperdiploid and tetraploid, with values of 19.6% (range 6–30%), 13.3% (5.6–26.2%) and 19.5% (9.8–34%) respectively ($P<0.05$).

Analysis of the effect on survival of clinical and cytometric parameters was undertaken for 81 RMS cases (Table II). Three clinical parameters (stage, tumour size and site), ploidy and S-phase were found to have a significant impact on survival. Overall survival by ploidy and S-phase are shown in a Kaplan–Meier analysis in Figures 3 and 4.

Five year survival rate in hyperdiploid RMS (DI 1.10–1.79) was 88.3% compared with 28.6% in tetraploid (DI $>1.80$), 44.4% in near-diploid (DI 1.0–1.09) and 58.3% in diploid tumours ($P = 0.0003$). Since there was no significant difference between diploid and near-diploid tumours, these two categories were combined (5 year survival 54.6%). Ninety-five per cent of children with an RMS and an S-phase below 14% were alive after 5 years compared with 50% with an S-phase above 14%. In the univariate analysis not only did tetraploid RMS have a significantly decreased survival compared with the hyperdiploid tumours, but also the outcome of the diploid/near-diploid category was significantly worse ($P = 0.0054$).

Multivariate analysis (Table III) could be performed on 59 cases with complete data and revealed that stage IV disease, tetraploidy, diploidy/near diploidy and S-phase ($>14\%$) were independently associated with significantly poorer survival. The threshold at 14% was selected by the stepwise analysis as the most discriminating variable.

When the analysis was repeated using only non-metastatic patients (stage I–III, 48 cases), hyperdiploidy was even more significant (relative hazard 12.24) and S-phase remained an independent prognostic indicator.

### Discussion

Ploidy has been evaluated and correlated with the outcome of treatment in several childhood malignancies. It is well

**Figure 1** Ploidy pattern.

**Table I** DNA ploidy in soft-tissue sarcomas

| | | *Ploidy pattern/S-phase* | | | | |
|---|---|---|---|---|---|---|
| *Source of tissue* | *Total* | *Diploid* | *Near-diploid* | *Hyperdiploid* | *Tetra-/ hypertetraploid* | *Median S-phase* |
| Rhabdomyosarcoma | 61 | 11 (18%) | 10 (15%) | 32 (49%) | 8 (17%) | 17.0% |
| Embryonal | 50 | 8 (16%) | 5 (10%) | 31 (62%) | 6 (12%) | 16.2%* |
| Alveolar | 11 | 3 (27%) | 5 (45%) | 1 (9%) | 2 (18%) | 22% * |
| PNET/EOE | 13 | 7 (57%) | 3 (21%) | 3 (21%) | 0 | 9.7% |
| Other sarcomas | 16 | 8 (50%) | 3 (19%) | 5 (31%) | 0 | 10.1% |

*Median S-phase significantly different ($P = 0.021$) between embryonal and alveolar RMSs.

**Table II** Univariate analysis of survival in 81 RMSs

| *Factor* | *No.* | *Five year survival (%)* | *P (log-rank test)* |
|---|---|---|---|
| Stage | | | |
| I | 15 | 90.9 | 0.0001 |
| II | 43 | 73.6 | |
| III | 10 | 85.7 | |
| IV | 13 | 10.8 | |
| Ploidy | | | |
| Diploid/near-diploid | 21 | 54.6 | 0.0003 |
| Hyperdiploid | 32 | 88.3 | |
| Tetraploid | 8 | 28.6 | |
| S-phase | | | |
| ⩾14% | 35 | 50.1 | 0.001 |
| <14% | 24 | 95.7 | |
| Site | | | |
| Limbs | 10 | 28.6 | 0.0008 |
| Others | 71 | 74.8 | |
| Tumour size | | | |
| <5 cm | 29 | 83.8 | 0.0218 |
| >5 cm | 52 | 60.3 | |

Age, sex, histology, surgical clearance (microscopically complete) and radiotherapy were not significant.

**Table III** Multivariate analysis in 59 RMSs (stepwise Cox's proportional hazard)

| *Variable* | *Factors* | *Poor prognosis feature* | *Adjusted relative hazard* | *95% confidence interval* |
|---|---|---|---|---|
| Stage | Stage IV *vs* others | Stage IV | 9.62 | 2.85–32.57 |
| Ploidy | Hyperdiploid *vs* others | Tetraploidy or diploidy/near diploidy | 6.91 | 1.70–27.24 |
| S-phase | ⩾14% *vs* <14% | >14% | 8.53 | 1.09–66.85 |

**Figure 2** Distribution of DNA index in soft-tissue sarcomas. **a**, Embryonal RMS (□) and alveolar RMS (■). **b**, PNET/EOE (□) and other sarcomas (■).

**Figure 3** RMS survival by ploidy.

**Figure 4** RMS survival by S-phase.

recognised that in subgroups of ALL (Look *et al.*, 1985) and neuroblastoma (Look *et al.*, 1984; Huddart *et al.*, 1992), hyperdiploid tumour stem lines seem to favour prognosis compared with their diploid or tetraploid counterparts, whereas this prognostic feature seems to be reversed in Wilms' tumour and most adult tumours (Douglass *et al.*, 1986; Schmidt *et al.*, 1986; Merkel *et al.*, 1987; Barrantes *et al.*, 1993). Data from other tumours such as medulloblastoma and hepatoblastoma are conflicting (Yasue *et al.*, 1989; Hata *et al.*, 1991; Zerbini *et al.*, 1993). There are only a few reports on the prognostic implication of DNA content in childhood STS and the results are not consistent (Boyle *et al.*, 1988; Molenaar *et al.*, 1988; Kowal-Vern *et al.*, 1990; Leuschner *et al.*, 1991; Shapiro *et al.*, 1991; Dias *et al.*, 1992).

The results in this study, so far the largest series evaluating this tumour group, confirm that ploidy has a significant and independent impact on outcome in rhabdomyosarcoma. Although stage IV disease is the most powerful predictor of outcome, tetraploidy/hypertetraploidy is strongly associated with an unfavourable prognosis, whereas hyperdiploidy (DI 1.10–1.80) is usually associated with a better outcome. The prognostic significance of the ploidy pattern was even more apparent in non-peripheral metastatic cases. In the univariate analysis diploid/near-diploid RMSs were associated with a significantly decreased survival compared with the hyperdiploid tumours. These results are similar to those of a study in St. Jude Children's Hospital (Shapiro *et al.*, 1991) and of the Intergroup Rhabdomyosarcoma Study (Pappo *et al.*, 1993), which found that hyperdiploidy also predicted a significantly favourable prognosis compared with diploidy/near diploidy. Similarly, tetraploidy was more often found in the alveolar subtype. We looked at four additional alveolar RMSs that were not included in this study because of age $>16$ years and found that three of them showed also tetraploidy, which increases the number with tetraploidy within the alveolar subtype to 33% compared with 12% in the embryonal RMSs. The discrepancies between these findings and those of some other studies (Leuschner *et al.*, 1991; Dias *et al.*, 1992), in which no correlation between DNA ploidy and overall survival could be found, may be explained by several factors: sample size, pathological classification, age distribution, flow cytometric analysis and precise ploidy definition.

In many studies, the definition of DNA ploidy does not extend beyond 'DNA diploid' and 'DNA aneuploid', and this pooling may mask the prognostic effect of different aneuploid types. This study suggests that there may be three distinct biological subtypes of RMS with different prognoses, namely diploid/near-diploid (DI = 1.0–1.09), hyperdiploid (DI = 1.10–1.80) and tetraploid/hypertetraploid (DI $>1.80$). In neuroblastoma it has been suggested that evolution of the tetraploid karyotype differs from the hyperdiploid, the former being caused by an endoreduplication from a primary diploid or near-diploid cell line (Kaneko *et al.*, 1987). A similar mechanism may be involved in RMS, as is suggested by our unpublished observations.

Flow cytometric measurement of formalin-fixed and paraffin-embedded tissue does, however, have its limitations and pitfalls. The intensity of the fluorochrome staining is dependent on the use of enzymes to break down covalent linkages between DNA and nuclear proteins caused by fixation (Hedley *et al.*, 1983), and the completeness of the digestive process in each sample may be difficult to gauge. Hence, the absolute fluorescence of the diploid populations can vary significantly from block to block, which precludes the use of external standards. Furthermore, hypodiploid cell populations cannot be distinguished in paraffin sections, but are assumed to be very rare. Nevertheless, several groups which have compared flow cytometric profiles from fresh and paraffin-embedded tissue (Hedley *et al.*, 1983; Frierson, 1988; Kallioniemi, 1988) have found a good correlation for the DNA index. Although a minor loss of quality in archival samples cannot be denied, and the techniques for preparing the sample may have to be tailored for different tissue groups, paraffin-embedded tissues are a convenient and reasonably accurate subject for DNA ploidy.

Of more concern is the variation in DNA ploidy that may occur in different samples of the same tumour. Intra-tumour variation in DNA ploidy is reported in the literature in up to 25% of some tumour types (Kallioniemi, 1988), and recently heterogeneity of DNA content was also reported in some RMSs (Dominici *et al.*, 1993). Multiple sampling for DI measurement is therefore recommended.

In several studies of adult malignancies, proliferative activity (S-phase) has been shown to be more powerful in predicting outcome than DNA index (Herman, 1992). However, major problems in estimating the S-phase are the overlap with normal host cells and the fact that the accuracy of the estimates is considerably reduced by large amounts of cell debris, especially in paraffin-embedded material. Comparisons of S-phase between different studies must be undertaken with caution since there are different models available for analysing the proliferative activity, and the measurement is also dependent on the computer software used. Nevertheless, in our population, we were able to demonstrate that S-phase had a significant impact on survival: only 1/24 children with RMS and an S-phase $<14\%$ died in this cohort of patients. If problems of accurately measuring the cell proliferative activity can be overcome, S-phase may become a useful prognostic factor with clinical significance.

The number of cases of non-rhabdomyosarcomatous soft-tissue sarcoma in this study was too small to allow firm conclusions to be made. Nevertheless Ewing's/PNET and other non-RMS STSs appear to have a different ploidy pattern and a significantly lower DNA index and proliferative activity than RMS. According to our results and a recent study of 19 patients with PNET (Swanson *et al.*, 1992) there is insufficient evidence that ploidy pattern may predict outcome in these rarer diagnoses.

We conclude that DNA content and S-phase in childhood STS have a significant prognostic impact. The biological behaviour of RMS can be divided at least into three different categories according to their DNA content. Hyperdiploid RMSs are associated with a more favourable prognosis than diploid/near-diploid or tetraploid/hypertetraploid tumours. Larger studies must be carried out to demonstrate this effect when children have received the same treatment protocol before DNA content and S-phase can be applied to the calculation of risk factors at diagnosis and used as an additional means of stratifying the treatment required.

We would like to thank Dr R. Pinkerton for permission to study patients under his care, Dr R. Carter for access to pathology material from patients treated at the Royal Marsden Hospital, Sutton, and Mr A. Brownhill, Mrs N. Costin-Kelly, Mrs C. Evans and Dr A.H. Cameron at the Children's Hospital, Birmingham, for technical assistance. This study was supported by a grant from ASTA Medica.

**References**

ANONYMOUS (1989). Prognostic factors in childhood rhabdomyosarcoma (editorial). *Lancet*, **ii**, 959–960.

BAISH, H., BECK, H.-P., CHRISTENSEN, I.J., HARTMANN, N.R., FRIED, J., DEAN, P.N., GRAY, J.W., JETT, J.H., JOHNSTON, D.A., WHITE, R.A., NICOLINI, C., ZEITZ, S. & WATSON, J.V. (1982). A comparison of mathematical methods for the analysis of DNA histograms obtained by flow cytometry. *Cell Tissue Kinet.*, **15**, 235–249.

BARRANTES, J.C., MUIR, K.R., TOYN, C.E., PARKES, S.E., CAMERON, A.H., MARSDEN, H.B., RAAFAT, F. & MANN, J.R. (1993). A thirty-year population based review of childhood renal tumours with an assessment of prognostic features including tumour DNA characteristics. *Med. Pediatr. Oncol.*, **21**, 24–30.

BOYLE, E.T., REIMAN, H.M., KRAMER, S.A., KELALIS, P.P., RAINWATER, L.M. & LIEBER, M.M. (1988). Embryonal rhabdomyosarcoma of bladder and prostate: nuclear DNA patterns studied by flow cytometry. *J. Urol.*, **140**, 1119–1121.

DIAS, P., KUMAR, P., MARSDEN, H.B., GATTAMANENI, H.R. & KUMAR, S. (1992). Prognostic relevance of DNA ploidy in rhabdomysarcomas and other sarcomas of childhood. *Anticancer Res.*, **12**, 1173–1178.

DOMINICI, C., PADULA, A., BASSO, G., BOSCO, S., CASTELLO, M.A., CECCAMEA, A., NINFO, V., TRAPASSO, E. & CARLI, M. (1993). DNA ploidy in rhabdomyosarcoma: is single site sampling enough for predicting outcome? SIOP XXV Meeting (abstract). *Med. Pediatr. Oncol.*, **21**, 599.

DOUGLASS, E.C., LOOK, A.T., WEBBER, B., PARHAM, D., WILLIAMS, J.A., GREEN, A.A. & ROBERSON, P.K. (1986). Hyperdiploidy and chromosomal rearrangements define the anaplastic variant of Wilm's tumor. *J. Clin. Oncol.*, **4**, 975–981.

FRIERSON, Jr, H.F. (1988). Flow cytometric analysis of ploidy in solid neoplasms: Comparison of fresh tissues with formalin fixed paraffin embedded specimens. *Hum. Pathol.*, **19**, 290–294.

HATA, Y., HAMADA, H., SASAKI, F., ISHIZU, H., OHMORI, K., UCHINO, J. & INOUE, K. (1991). Flow cytometric analysis of the nuclear DNA content of hepatoblastoma. SIOP XXIII Meeting (abstract). *Med. Pediatr. Oncol.*, **19**, 348.

HEDLEY, D.W., FRIEDLANDER, M.L., TAYLOR, I.W., RUGG, C.A. & MUSGROVE, E.A. (1983). Method for analysis of cellular DNA content of paraffin-embedded pathological material using flow cytometry. *J. Histochem. Cytochem.*, **31**, 1333–1335.

HERMAN, C.J. (1992). Cytometric DNA analysis in the management of cancer. *Cancer*, **69** (Suppl.), 1553–1556.

HUDDART, S.N., MUIR, K.R., PARKES, S., MANN, J.R., STEVENS, M.C.G., RAAFAT, F. & SMITH, K. (1993). Prognostic significance of DNA ploidy and proliferative activity in neuroblastoma – a retrospective study. *J. Clin. Pathol.*, **46**, 1101–1104.

KANEKO, Y., KANDA, N., MASEKI, N., SAKURAI, M., TSUCHIDA, Y., TAKEDA, T., OKABE, I. & SAKURAI, M. (1987). Different karyotypic patterns in early and advanced stage neuroblastomas. *Cancer Res.*, **47**, 311–318.

KALLIONIEMI, P.-P. (1988). Comparison of fresh and paraffin-embedded tissue as starting material for DNA flow cytometry and evaluation of intratumor heterogeneity. *Cytometry*, **9**, 164–169.

KOWAL-VERN, A., GONZALEZ CRUSSI, F., TURNER, J., TRUJILLO, Y.P., CHOU, P., HERMAN, C., CASTELLI, M. & WALLOCH, J. (1990). Flow and image cytometric DNA analysis in rhabdomyosarcoma. *Cancer Res.*, **50**, 6023–6027.

LEUSCHNER, I., SCHMIDT, D., MÖLLER, R. & HARMS, D. (1991). DNA ploidy and nucleolar organizer regions in rhabdomyosarcoma. SIOP XXIII Meeting (abstract). *Med. Pediatr. Oncol.*, **19**, 350.

LOOK, A.T., HAYS, F.A., NITSCHKE, R., MCWILLIAMS, N.B. & GREEN, A.A. (1984). Cellular DNA-content as a predictor of response to chemotherapy in infants with unresectable neuroblastoma. *N. Engl. J. Med.*, **311**, 231–235.

LOOK, A.T., ROBERTSON, P.K., WILLIAMS, D.L., RIVERA, G., BOWMAN, W.P., PUI, C.-H., OCHS, J., ABROMOWITCH, M., KALWINSKY, D., DAHL, G.V., GEORGE, S. & MURPHY, S.B. (1985). Prognostic importance of blast cell DNA content in childhood acute lymphoblastic leukemia. *Blood*, **65**, 1079–1086.

MAURER, H.M., GEHAN, E.A., BELTANGADY, M., CRIST, W., DICKMAN, P.S., DONALDSON, S.S., FRYER, C., HAMMOND, D., HAYS, D.M., HERRMANN, J., HEYN, R., MORRIS JONES, P., LAURENCE, W., NEWTON, W., ORTEGA, J., RAGAB, A.H., RANEY, R.B., RUYMANN, F.B., SOULE, E., TEFFT, N., WEBBER, B., WIENER, E., WHARAN, M. & VIETTI, T.J. (1993). The Intergroup Rhabdomyosarcoma Study II. *Cancer*, **71**, 1904–1922.

MERKEL, D.E., DRESSLER, L.G. & MCGUIRE, W.L. (1987). Flow cytometry, cellular DNA content, and prognosis in human malignancy. *J. Clin. Oncol.*, **5**, 1690–1703.

MOLENAAR, W.M., DAM-MEIRING, A., KAMPS, W.A. & CORNELISSE, C.J. (1988). DNA-aneuploidy in rhabdomyosarcomas as compared with other sarcomas of childhood and adolescence. *Hum. Pathol.*, **19**, 573–579.

PAPPO, A.S., CRIST, W.M., KUTTESCH, J., ROWE, S., ASHMUM, R.A., MAURER, H.M., NEWTON, W.A., ASMAR, L. LUO, X. & SHAPIRO, D.N. (1993). Tumor cell DNA content predicts outcome in children and adolescents with clinical group III embryonal rhabdomyosarcoma. *J. Clin. Oncol.*, **11**, 1901–1905.

RAGAB, A., GEHAN, E.A., MAURER, H.M., ORTEGA, J., WIENER, E., NEWTON, W., WHARAM, M. & MORRIS-JONES, P. (1992). Intergroup Rhabdomyosarcoma Study (IRS). III. Preliminary report of the major results. ASCO annual meeting. *Proc. Am. Soc. Clin. Oncol.*, **11**, 363.

RODARY, C., FLAMANT, F. & DONALDSON, S.S. (FOR THE SIOP-IRS COMMITTEE) (1989). An attempt to use a common staging system in rhabdomyosarcoma: a report of an international workshop initiated by the International Society of Pediatric Oncology (SIOP). *Med. Pediatr. Oncol.*, **17**, 210–215.

RODARY, C., GEHAN, E.A., FLAMANT, F., TREUNER, J., CARLI, M., AUQUIER, A. & MAURER, H. (1991). Prognostic factors in 951 non-metastatic rhabdomyosarcomas in children: a report from the International Rhabdomyosarcoma Workshop. *Med. Pediatr. Oncol.*, **19**, 89–95.

SCHMIDT, D., WIEDEMANN, B., KEIL, W., SPRENGER, E. & HARMS, D. (1986). Flow cytometric analysis of nephroblastomas and related neoplasms. *Cancer*, **58**, 2494–2500.

SCOTT, N., CROSS, D., PLUMB, M.I., DIXON, M.F. & QUIRKE, P. (1992). An investigation of different methods of cell cycle analysis by flow cytometry in rectal cancer. *Br. J. Cancer*, **65**, 8–10.

SHAPIRO, D.N., PARHAM, D.M., DOUGLASS, E.C., ASHMUM, R., WEBBER, B.L., NEWTON, W.A., HANCOCK, M.L., MAURER, H.M. & LOOK, A.T. (1991). Relationship of tumour-cell ploidy to histologic subtype and treatment outcome in children and adolescents with unresectable rhabdomyosarcoma. *J. Clin. Oncol.*, **9**, 159–166.

STEVENS, M.C.G., FLAMANT, F. & REY, A. (1991). SIOP mesenchymal malignant tumour (MMT) 1989 study. *Med. Pediatr. Oncol.*, **19**, 435.

SWANSON, P.E., JASZCZ, W., NAKHLEH, R.E., KELLY, D.R., DEHNER, L.P. & LAUREN, V. (1992). Peripheral primitive neuroectodermal tumors. A flow cytometric analysis with immunohistochemical and ultrastructural observations. *Arch. Pathol. Lab. Med.*, **116**, 1202–1208.

YASUE, M., TOMITA, T., ENGELHARD, H., GONZALEZ-CRUSSI, F., MCLONE, D.G. & BAUER, K.D. (1989). Prognostic importance of DNA ploidy in medulloblastoma of childhood. *J. Neurosurg.*, **70**, 385–391.

ZERBINI, C., GELBER, R.D., WEINBERG, D., SCALLAN, S.E., BARNES, P., KUPSKY, W., SCOTT, R.M. & TARBELL, N.J. (1993). Prognostic factors in medulloblastoma, including DNA ploidy. *J. Clin. Oncol.*, **11**, 616–622.